# Quantitative redox imaging biomarkers for studying tissue metabolic state and its heterogeneity

**DOI:** 10.1142/S179354581430002X

**Published:** 2014-03

**Authors:** He N Xu, Lin Z Li

**Affiliations:** Department of Radiology, Britton Chance Laboratory of Redox Imaging, Johnson Research Foundation, Department of Biochemistry and Biophysics, Perelman School of Medicine, University of Pennsylvania, Philadelphia, PA, USA

**Keywords:** Tumor progression, mitochondria, metastasis, metastatic potential, pre-malignancy, NADH, Fp, FAD, redox ratio

## Abstract

NAD^+^/NADH redox state has been implicated in many diseases such as cancer and diabetes as well as in the regulation of embryonic development and aging. To fluorimetrically assess the mitochondrial redox state, Dr. Chance and co-workers measured the fluorescence of NADH and oxidized flavoproteins (Fp) including flavin-adenine-dinucleotide (FAD) and demonstrated their ratio (i.e. the redox ratio) is a sensitive indicator of the mitochondrial redox states. The Chance redox scanner was built to simultaneously measure NADH and Fp in tissue at submillimeter scale in 3D using the freeze-trap protocol. This paper summarizes our recent research experience, development and new applications of the redox scanning technique in collaboration with Dr.Chance beginning in 2005. Dr. Chance initiated or actively involved in many of the projects during the last several years of his life. We advanced the redox scanning technique by measuring the nominal concentrations (in reference to the frozen solution standards) of the endogenous fluorescent analytes, i.e., [NADH] and [Fp] to quantify the redox ratios in various biological tissues. The advancement has enabled us to identify an array of the redox indices as quantitative imaging biomarkers (including [NADH], [Fp], [Fp]/([NADH] + [Fp]), [NADH]/[Fp], and their standard deviations) for studying some important biological questions on cancer and normal tissue metabolism. We found that the redox indices were associated or changed with (1) tumorigenesis (cancer versus non-cancer of human breast tissue biopsies); (2) tumor metastatic potential; (3) tumor glucose uptake; (4) tumor p53 status; (5) PI3K pathway activation in premalignant tissue; (6) therapeutic effects on tumors; (7) embryonic stem cell differentiation; (8) the heart under fasting. Together, our work demonstrated that the tissue redox indices obtained from the redox scanning technique may provide useful information about tissue metabolism and physiology status in normal and diseased tissues. The Chance redox scanner and other redox imaging techniques may have wide-ranging potential applications in many fields, such as cancer, diabetes, developmental process, mitochondrial diseases, neurodegenerative diseases, and aging.

## 1. Introduction

The mitochondrial redox state as represented by mitochondrial NAD^+^/NADH indicates metabolic functions of the mitochondria and plays important roles in many diseases such as cancer^1^ and diabetes^2^ as well as in the regulation of embryonic development^3^ and aging.^4^ Dr. Chance pioneered the optical determination of the mitochondrial redox state by measuring the fluorescence from mitochondrial NADH and oxidized flavoproteins Fp including FAD and demonstrated that the redox ratio is a sensitive indicator of the mitochondrial redox state.

NADH and Fp become the intrinsic metabolic biomarkers with a long research history. NAD^+^ molecule and its absorption at 340 nm were first identified by Otto Warburg in 1930s.^5^ The fluorescence of NADH was also first observed by Warburg.^6^ In 1950s, Chance *et al.* used the characteristic absorption of NADH to study the respiratory enzymes in the mitochondria. In a series of classical papers published in 1955,^7–11^ Chance and Williams defined the metabolic states of the isolated mitochondria for the first time, and correlated these metabolic states to the oxidation-reduction levels of the respiratory enzymes. In 1957, Duysens and Amesz reported the first observation of NADH fluorescence in cells.^12^ In 1958, Chance and Ralt-scheffsky first reported NADH fluorescence from the isolated mitochondria and defined three redox states with State 4 having the highest and State 2 having the lowest NADH fluorescence, respectively.^13^ Since then Dr. Chance and his colleagues started extensive investigations on fluorescently monitoring NADH and the roles NADH play in bioenergetics. A landmark paper on monitoring NADH fluorescence *in vivo* in the brain and kidney was published in 1962 by Chance *et al.*^14^ The techniques for *in vivo* monitoring NADH have been further developed by Chance and his colleagues from animal models to patients.^15^

The optical properties of Fp were discovered and characterized by Warburg *et al.* in 1930s to have the absorption peaks at 375 nm and 450 nm.^16–18^ In 1966, Chance and Schoener^19^ obtained the excitation and emission spectra of the mitochondria, cells, and tissues and identified a measureable component of the flavin groups that are responsive to the mitochondrial redox state change. Oxidization causes an increase in the fluorescence of these flavins. Chance *et al.* characterized the sites of multiple Fp (including an electron transport Fp component) in the electron transport chain of the mitochondria from 1967 to 1968.^20–22^ Later the Fp fluorescence signals were confirmed to have three contributing parts: lipoamide dehydrogenase associated Fp, electron transport Fp, and dithiniote-reducible-only Fp.^23–26^ A number of studies have shown NADH and Fp signals originated mainly from the mitochondria.^27–30^ Further studies were conducted by the Chance group to simultaneously monitor NADH and Fp as intrinsic fluorescent sources *in vivo* using a time-sharing fluorometer.^31^ From 1970s to early 1980s, the Chance lab also developed a time-sharing four-channel cryogenic fluorescence imager,^32,33^ i.e., the Chance redox scanner that can simultaneously measure NADH, Fp and the tissue redox state *ex vivo* at a 3D resolution of 50 × 50 × 20 *μ*m^3^.

Numerous studies have shown that NADH, Fp and the redox ratio are sensitive to cellular metabolic state. For example, Chance *et al.* showed that the redox ratio Fp/NADH or NADH/Fp may provide a surrogate biomarker for the mitochondrial oxidation-reduction state imposed by metabolites in the isolated mitochondria and the ratios were insensitive to blood hemodynamic factors such as red blood cell content.^34^ Ozawa *et al.* showed in 1992 that the redox ratio Fp/NADH may be a surrogate biomarker for NAD^+^/NADH by correlating the redox ratio of liver specimens linearly with the redox potential NAD^+^/NADH as measured by the concentration ratio of arterial blood ketone bodies.^35^ The changes of the redox distribution of the liver caused by ischemia were quantified using Fp/NADH and its standard deviation.^36^ Normalized redox ratio Fp/(Fp+NADH) and NADH/ (Fp+NADH) were also used later.^27,37,38^ Auto-fluorescence of NADH and Fp and the redox ratios have been extensively used to study tissue redox state and its heterogeneity in organs such as brain, liver, heart,^36,38–42^ and cancer.^27,43–48^

While other groups also contributed to the studies of NADH and Fp as intrinsic fluorescent biomarkers, Dr. Chance and coworkers were certainly leading the field for many years. In his unpublished autograph, Dr. Chance regarded the measurement of the intrinsic fluorescence of NADH and Fp from the mitochondria as “perhaps the most important discovery of my career because for the first time we could obtain optical signals from living mitochondrial tissues.” Dr. Chance’s pioneer work on assessing the mitochondrial redox state by optical methods inspired numerous scientific efforts to investigate its importance with various model systems and more advanced instrumentations, such as two photon microscopy^49^ which may non-invasively probe tissue redox state to a depth less than 1 mm.

The redox scanning technique developed by Chance *et al.* remains to be one of the most effective ways of imaging the redox state in deep tissue. The major advantages of this technique include: (1) preservation of the *in vivo* metabolic state by snapfreezing tissue in liquid nitrogen so that the *ex vivo* scanning results can represent the metabolic state under the *in vivo* conditions; (2) 10 folds or more enhancement of NADH and Fp fluorescence signals by imaging at the low temperature of liquid nitrogen; (3) imaging the deep tissue metabolic heterogeneity by its 3D mapping capability with submillimeter resolution.

This article summarizes our research on redox scanning in collaboration with Dr. Chance during the last six years of his life and the work we continued to carry out. In early 2005, Dr. Lin Li and Ms. Tuoxiu Zhong initiated a redox scanning test on two melanoma xenografts with different aggressiveness. The imaging data revealed distinct differences between the metastatic and the relatively indolent melanomas. Drs. Glickson and Chance were very excited to see the big difference, and Dr. Glickson described it as the difference between “day and night”. They immediately realized the clinical potential of this result, and jump-started the collaboration between the Glickson lab and the Chance lab on imaging the metastatic potential using the melanoma mouse models. In 2006 leaving for Singapore, Dr. Chance designated Dr. Li to direct the redox scanning lab along with the support from Dr. Glickson and later from Dr. Ravinder Reddy. Dr. He Xu joined the redox scanning study in the summer of 2007. Significant amount of data on tumor models, clinical specimens, and normal tissues have been produced in the past six years. These data demonstrated that the redox imaging biomarkers obtained by the redox scanning are useful for studying tissue metabolic state and its heterogeneity in both normal health and diseases.

## 2. The Further Development of the Redox Scanning Technique

### 2.1. Quantification of NADH and Fp concentrations in reference to the standards

In 2007, we realized that for the quantitative redox measurements, a reliable calibration procedure was needed to compare redox scanning results from different samples or the same sample scanned at different time. We decided to use frozen NADH and FAD solutions as the reference standards that were placed adjacent to the tissue samples. This idea did not become a reality until we used a short piece of Teflon tubing with one end being sealed by a hot soldering gun to contain the standard solution. We first identified the linear dynamic range of the NADH and Fp channels of the Chance redox scanner. A series of solution standards of both NADH and FAD at various concentrations was prepared using 10 mM tris-HCl (pH = 7) as the dilution solvent. By scanning the snap-frozen standards we obtained two sets of calibration curves of NADH and FAD at different voltages of the photo-multiplier tube (PMT), respectively.^50,51^ These enabled us to quantify the nominal concentrations of NADH and Fp in the tissue samples in reference to the corresponding standards after subtracting the background fluorescence signals on the mounting medium and the standards intensity.

However, there are still limitations to the quantification method we developed. For example, if Hg arc lamp power varied in large degree within the time frame of a scanning session, different quantitative results could arise when scanning the same tissue section. This can be avoided by igniting the lamp ~ 60 min before use so that the lamp becomes stable. In addition to the instrument factors, such as the performance and stability of the light source and the PMT detector as well as the electronics, which have been taken care of by our calibration procedures, we later discovered that different fiber-optic light guide yielded different calibration curves and linear dynamic range. As the light guide uses quartz and regular glass for transmitting the excitation light and emission light, respectively, and epoxy for gluing the fiber bundles, fluorescence can be generated from both the fiber and the epoxy. The intensity of the fluorescence varies with the materials used to construct the fiber-optical light guide. Fortunately the influence of these fluorescence contaminations is controllable with the background removal and calibration procedures, and it does not prevent us from developing autofluorescence signals as the biomarkers. Whenever a new light guide is used, a new set of calibration curves should be generated to determine the linear dynamic range in order to determine the concentration of the reference standards for tissue embedding. Apart from these fluorescence contaminations, several other factors may also affect the fluorescence emission signals. Due to imperfection of the scanning surfaces, small variation of the distance between the fiberoptic guide tip and the imaging plane (usually kept at 70 μm) may influence the signal. Other factors include light absorption, scattering, and the penetration depths, which all could vary among tissues and between the tissues and the reference standards. Therefore the absolute quantification of NADH and Fp concentrations is difficult, but the nominal quantification appears to be sufficient for providing the quantitative biomarkers to differentiate the redox states between normal and diseased tissues.

### 2.2. Development of the redox parameters for quantitatively addressing tissue heterogeneity

In addition to its genetic heterogeneity, tumor tissue is also metabolically heterogeneous. For example, cross-section of a FaDu tumor xenograft exhibited large spatial heterogeneity in pO_2_, glucose concentration, and lactate concentration and there is no apparent correlation among these metabolic parameters.^52^ We observed large spatial distribution of the redox indices (NADH, Fp, and their ratios) in aggressive solid tumors, such as melanomas,^44^ breasttumors,^47,53,54^ prostate tumors,^55^ and colon tumors.^45^ We also observed significant heterogeneity in the redox state of human breast cancer biopsies from the same patient.^56,57^ Even premalignant tissue displays significant heterogeneity in the redox state compared to the normal one, as we showed in the PTEN-null pancreatic tissue in transgenic mouse model.^46,58^ Often times, the intra-tumor heterogeneity in the redox state is larger than the inter-tumor heterogeneity. For example, the Fp redox ratio, Fp/(NADH + Fp) of the aggressive breast tumors can vary ~ 50% within the same tumor while the inter-tumor difference in the same index varies ~ 20%.^47,53,54^

However, the commonly used global averaging quantification (averaging over an entire tumor/ sample, resulting in loss of spatial variation information) is insensitive to or largely ignores intra-tissue heterogeneity and only reflects the inter-tumor or inter-sample variation. The global averaging approach may fail to reveal the statistical difference of two means due to large intra-tumor variations. For example, we were not able to differentiate among the five melanoma lines spanning a full range of metastatic potential^44^ or the two breast tumor lines^47^ of different aggressiveness by the global-averaged Fp redox ratio. The significant redox shift and redox heterogeneity caused by pancreas-specific PTEN-deletion was not seen by global averaging,^46,58^ either.

Our research experience indicated that in order to differentiate among tumors with different aggressiveness or discriminate (pre)malignancy from normal tissues, we should employ certain intra-sample heterogeneity indices. Compared to the normal tissues, a wide histogram with a large standard deviation of the redox indices is a common feature of both tumor and premalignant tissue. A well-separated bi-modal distribution of the redox indices with larger standard deviations (SD) of the means is a common feature of the more aggressive tumors, and a relatively narrower histogram with smaller SD is associated with less aggressiveness. The approaches we have taken to address the heterogeneity include either using the standard deviations of the histograms of the redox indices as a measure of heterogeneity, or fitting Gaussian functions to the histograms of the redox indices to obtain the mean values, amplitudes, and widths of the Gaussian distributions.^58^ Once the parameters are obtained, we then use tissue depth as a covariate in the statistical analysis of multiple tissue sections. In the future, we may alternatively treat the multiple imaging sections of a specimens as repeated measurements and employ the linear mixed-effect model to perform statistical analysis of the imaging data. These approaches largely take care of the intra-tumor heterogeneity and are more effective for discriminating cancer from normal tissue and predicting tumor metastatic risk. We will discuss this more in Sec. 3.1.

In summary, we learned that to fully characterize and classify the heterogeneous tissue samples such as cancer and pre-cancer, we should interpret the metabolic imaging data using certain intra-sample heterogeneity indices to evaluate their biological significance.

## 3. Biomedical Applications of the Chance Redox Scanner

### 3.1. Redox scanning of cancer and premalignant tissue

#### 3.1.1. Redox state abnormality in clinical breast cancerous tissue

Visual examination of the histological staining of biopsy samples by the pathologists has been the standard clinical practice to differentiate between cancer and non-cancer and grade tumors. However, the histological assay is limited in predicting cancer prognosis in general. Both staining and visual evaluations are time-consuming and cumbersome for a large number of tissue slides in practice. Visual examination may also be subject to variations from different or even the same pathologist. In addition, current pathological evaluation may overlook the fact that the histopathologically same biopsies may have a different metabolic state and/or heterogeneity patterns *in vivo*. As alteration of tissue metabolism has been recognized as one of the characteristics of malignancy^59–61^ and the autofluorescence-based redox indices we developed are sensitive to tissue metabolism, it is of our great interest to translate the redox scanning technique to clinical specimens. In principle redox scanning can be implemented in the clinic for real time surgery monitoring and diagnosis.

In 2010 we started our collaboration with Dr. Julia Tchou, who provided us the breast tissue core needle biopsies (or core biopsies) taken from her patients. The redox indices readily distinguished cancer from non-cancer biopsies from three female breast cancer patients and the cancerous tissues had up to 10-fold higher Fp signal and were more oxidized with the Fp redox ratio being ~ 50–70% higher than the normal counterparts.^56,57^ Dr. Chance was extremely excited by the results which also puzzled him a great deal. He spent sleepless nights thinking about the metabolisms of the core biopsies, asked many questions on the details of how the experiments were performed. Once he confirmed all the details, he provided his deep insight into the underlying biochemistry and was eager to publish the results. On August 3rd in 2010, he wrote: “Biochemistry of core biopsies has had little diagnostic value because the small anaerobic sample availability and the lack of biochemical identification of cancer. A novel approach to the biochemistry of biopsies is afforded by the redox scanner which requires only a small tissue sample (1 mm in two dimensions)” for the 1st draft of the manuscript.

**Fig. 1.**
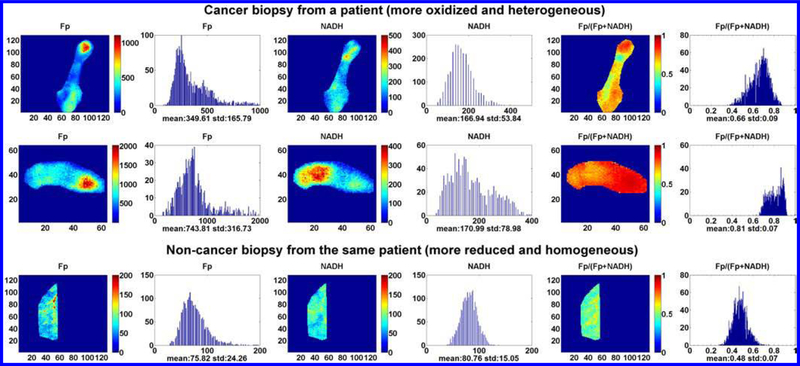
The typical redox images of the cancerous and non-cancerours breast tissue biopsies from a patient. From left to right: Fp, NADH, and the Fp redox ratios images and the corresponding histograms. The figure is reproduced with permission from Ref. 57.

In addition to the much higher Fp signals, the cancerous tissues were significantly more heterogeneous in the redox state. Also, NADH intensity was about two-three times higher in the tumor tissue but no statistical difference found between the tumor and the normal tissues due to the large standard deviation of NADH in tumors. Even from the same patient, NADH of biopsies sampled from different locations of the tumor could vary by five times (45–221 *μ*M, nominal concentration). As an example, Fig. 1 shows the typical redox images and their corresponding histograms. A separate manuscript is being prepared to report the redox scanning results from larger patient numbers and the correlation of the redox indices with the clinical diagnostic parameters.

We also compared the redox state of the breast tumor xenografts with that of the mouse leg muscles (see next section). The result is consistent with the clinical findings aforementioned. The leg muscles were more reduced and less heterogeneous in their redox state.^37^ This is also shown by our unpublished data.

#### 3.1.2. The association of tumor metastatic risk with the redox indices in mouse models

Our long term goal was to identify the metabolic imaging biomarkers for predicting tumor metastatic potential and prognosis. Metastasis accounts for 90% of cancer death. There are no clinically reliable methods or markers for predicting tumor metastatic risk in general. It is common for a cancer patient to be either over or under diagnosed and treated. Considering the heterogeneous nature of tumors, quantitative molecular imaging biomarkers for differentiating tumors of different metastatic potentials are highly desired. Here we review our main results from redox imaging of the human melanomas and breast tumors in mouse models.

##### 3.1.2.1. Melanomas

**Fig. 2.**
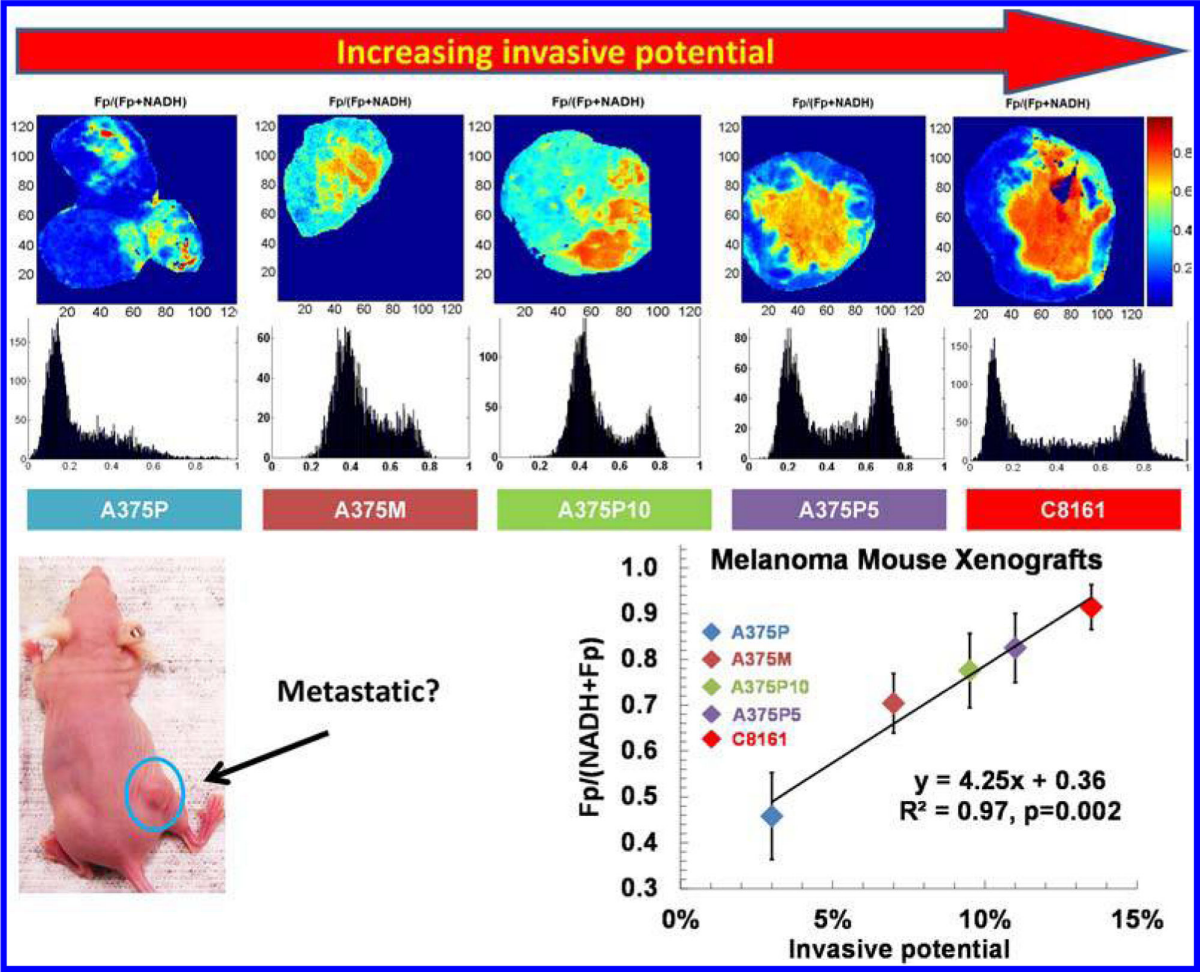
The typical Fp redox ratio images and their corresponding histograms of the five melanoma lines (A375P, A375M, A375P10, A375P5, and C8161) and the positive linear correlation between the Fp redox ratio and tumor invasive potential. The Fp redox ratios in the redox core (the right peaks in the histograms) were plotted against the invasive potentials of the five cell lines measured by the Boyden chamber method. The figure is reproduced with permission from Ref. 44.

The initial test was performed using the mouse xenografts of two human melanoma lines, i.e., the less metastatic/invasive A375P and the highly metastatic/invasive C8161.^37^ The highly metastatic tumors had localized regions with a relatively higher Fp redox ratio compared to surrounding regions. We define the more oxidized region as the redox core, which may or may not co-localize with tumor central region. The relatively more reduced region is defined as the redox rim, which may or may not colocalize with tumor peripheral. The less metastatic tumors exhibited a relatively more reduced and relatively uniform redox ratio distribution similar to the redox rim of the highly metastatic tumors. When three extra lines of melanoma with intermediate invasive potentials were added to the study, the Fp redox ratios of the redox core surprisingly had a positive linear correlation with the invasive potentials with *R*^2^ = 0.97 and *p =* 0.002 (Fig. 2).^44^ Histological studies identified the existence of many viable cells in the oxidized cores of the aggressive tumors.^62^ However, a much poorer correlation (*R*^2^ = 0.63, *p* = 0.1) was found if the redox ratio averaging was taken for the entire tumor. The results indicate that the redox state of the redox core seems to be a more effective biomarker for predicting tumor metastatic risk.

##### 3.1.2.2. Breast tumors

Encouraged by the melanoma imaging results, we expanded the redox scanning study to breast tumors starting in the spring of 2008. Initially the relatively indolent MCF-7 and the aggressive MDA-MB-231 tumors were grown to 6–10 mm in diameter and subjected to the redox scanning. Similar to the melanoma results, the MDA-MB-231 tumors exhibited a much more oxidized redox core while the relatively indolent MCF-7 tumors were largely uniform in the redox state; the globally averaged redox indices failed to differentiate them but the indices of the redox core easily discriminate these two lines. We discussed the data with Dr. Chance when he returned to Philadelphia from Taiwan in the summer of 2009. He was impressed with the results and suggested to publish the findings. He carefully reviewed the manuscript draft and gave detailed explanations on bioenergetics such as how the redox ratio was related to free energy, which were added to the relevant sections of the manuscript. During our discussion with Dr. Chance, the Warburg effect/hypothesis was brought up, which led us to carefully examine the original papers both Warburg and Dr. Chance published in Science in 1950s.^63,64^ Dr. Chance’s data clearly showed that the same cancer cells Warburg used for his discovery of aerobic glycolysis (the Warburg effect) did not have suppressed mitochondrial function (the Warburg hypothesis). We added a section of the discussion on the Warburg effect to the manuscript published in 2010.^47^

**Fig. 3.**
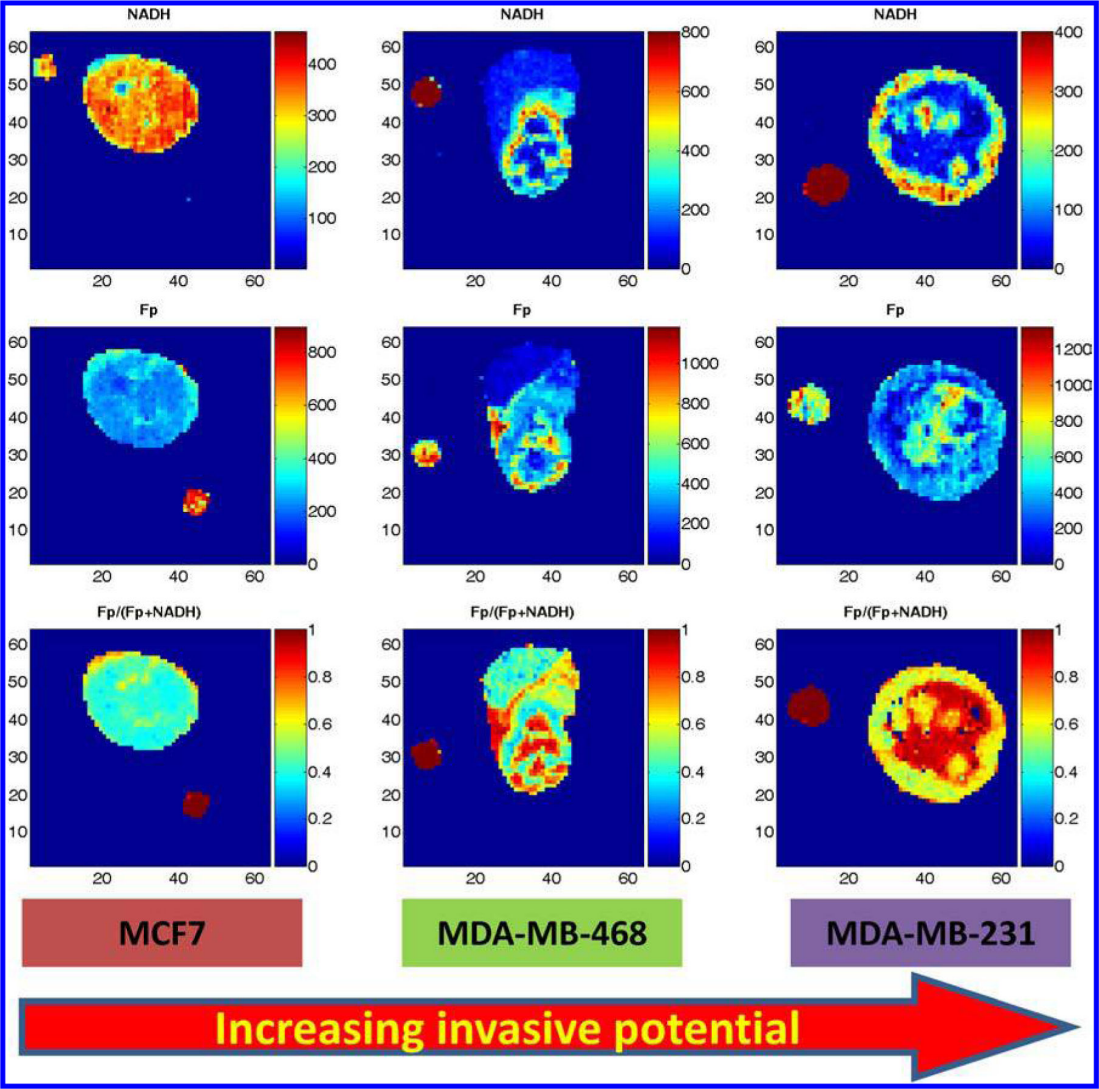
The typical redox images (NADH, Fp, the Fp redox ratio) of three breast cancer xenografts with increasing invasive potential MCF7<MDA-MB-468<MDA-MB-231. The figure is reproduced with permission from Ref. 54.

We then decided to add another breast tumor line, MDA-MB-468 to the study. This line is less invasive than MDA-MB-231 but more invasive than MCF-7. Figure 3 shows the typical redox images of the three lines. Unlike MDA-MB-231 tumors, the first few MDA-MB-468 xenografts exhibited interesting redox state distribution patterns that were not co-localized with the spatial core and rim, but with rather scattered or patchy redox cores (we still call them the redox cores as previously defined in Sec. 3.1.2, i.e., the areas with higher Fp redox ratios). We then became interested in learning how the redox cores distribute in three dimensions. Scanning section by section we exhausted the entire tumors and obtained quantitative distribution of the redox indices in three dimensions. After carefully looking through the scanning data, Dr. Chance was quite pleased to point out: “It is good to know that using the redox data from three to five sections to represent the entire tumor’s redox state was not far off.” He again happily co-authored the preliminary short report on the findings.^54^ We discovered that the Fp redox ratio of MDA-MB-468 tumors falls between that of MCF-7 and MDA-MB-231 tumors. We were pleased to see that the Fp redox ratios of these three mouse xenografts also have positive linear correlation with the invasive potentials^104^ (a full manuscript is under preparation). We also realized that the reported redox ratios of these three lines *in vitro*^65^ are consistent with our findings on the mouse xenografts of these tumor lines.

It is known that certain hypoxic-ischemic tissue yields higher mitochondrial NADH and lower Fp, i.e., the more reduced mitochondrial redox state, particularly for acute hypoxia.^66,67^ One could expect that the tumor central region may be relatively more reduced due to its poorer blood supply than the periphery and thus somewhat more hypoxic than the periphery. However, we observed a more oxidized mitochondrial redox state in the central regions of the aggressive tumors. This paradoxical finding may be understood by considering the degree of hypoxia that could affect the mitochondrial redox state in tumor tissues. It has been noted in the literature that greater than 4–8 *μM* of oxygen would saturate OXPHOS; and only when oxygen concentration surrounding the mitochondria is below the critical level of 1 *μ*M, apparent change in OXPHOS and electron transfer chain (ETC) state can be seen in the mitochondrial metabolism of tumor cells.^68^ However, it is still possible that prolonged hypoxia may affect the mitochondrial metabolism indirectly by altering the expression of certain mitochondrial enzymes. Imaging both oxygen level and the mitochondrial redox state of the same tumor may provide some insights into the paradoxical findings.

The tumor metastatic potential involves multifactors. Our research data indicate the mitochondrial redox state might be an important factor for the metastatic process. A commonly held belief is that cancer cells need angiogenesis to grow and metastasize and that blocking tumor angiogenesis may suppress tumor metastasis. However, such inhibition can actually enhance metastasis, which is shown to be the case for some anti-angiogenesis therapies in animal models.^69^

In order to understand the histological basis of those localized regions with more oxidized redox states in the aggressive tumors, we stained the tumor tissue sections of melanoma xenografts.^62^ The H&E staining revealed that the cells in these regions appeared pinkish, indicative of necrosis. However, the DAPI staining clearly showed the existence of many intact nuclei and the TUNEL staining indicated a low level of cell death. Further work in collaboration with Drs. Julian Lum, Ravi Amaravadi, and Xiaohong Ma demonstrated the more aggressive melanomas have a higher level of autophagy, which is a cell survival mechanism under nutritional and redox stress.^70^ Although this provides a plausible mechanism for the survival of cancer cells in the oxidized cores, the relationship among tumor redox state, autophagy, and metastatic potential remains to be explored.

#### 3.1.3. The correlation between the redox state, glucose uptake, ROS, and genetics

As we observed that the more aggressive tumors have localized regions with a more oxidized redox state, the metabolic state and its heterogeneity in these localized oxidized regions were of interest to us. Metabolic heterogeneity of cancer tissues has been studied in many aspects, such as the spatial distribution of glucose uptake, lactate release, and ATP.^71,72^ We imaged glucose uptake in individual breast tumor xenografts by simultaneously scanning Fp, NADH, and Pyro-2DG (a near-IR fluorescent glucose analog whose signal represents the glucose uptake). The imaging data showed that higher glucose uptake occurred in the periphery and some areas in the oxidized core also had significant Pyro-2DG uptake.^73^ Further analysis of the data showed a positive linear correlation between the NADH redox ratio (NADH/ (Fp + NADH)) and Pyro-2DG (Fig. 6).^53^

**Fig. 4.**
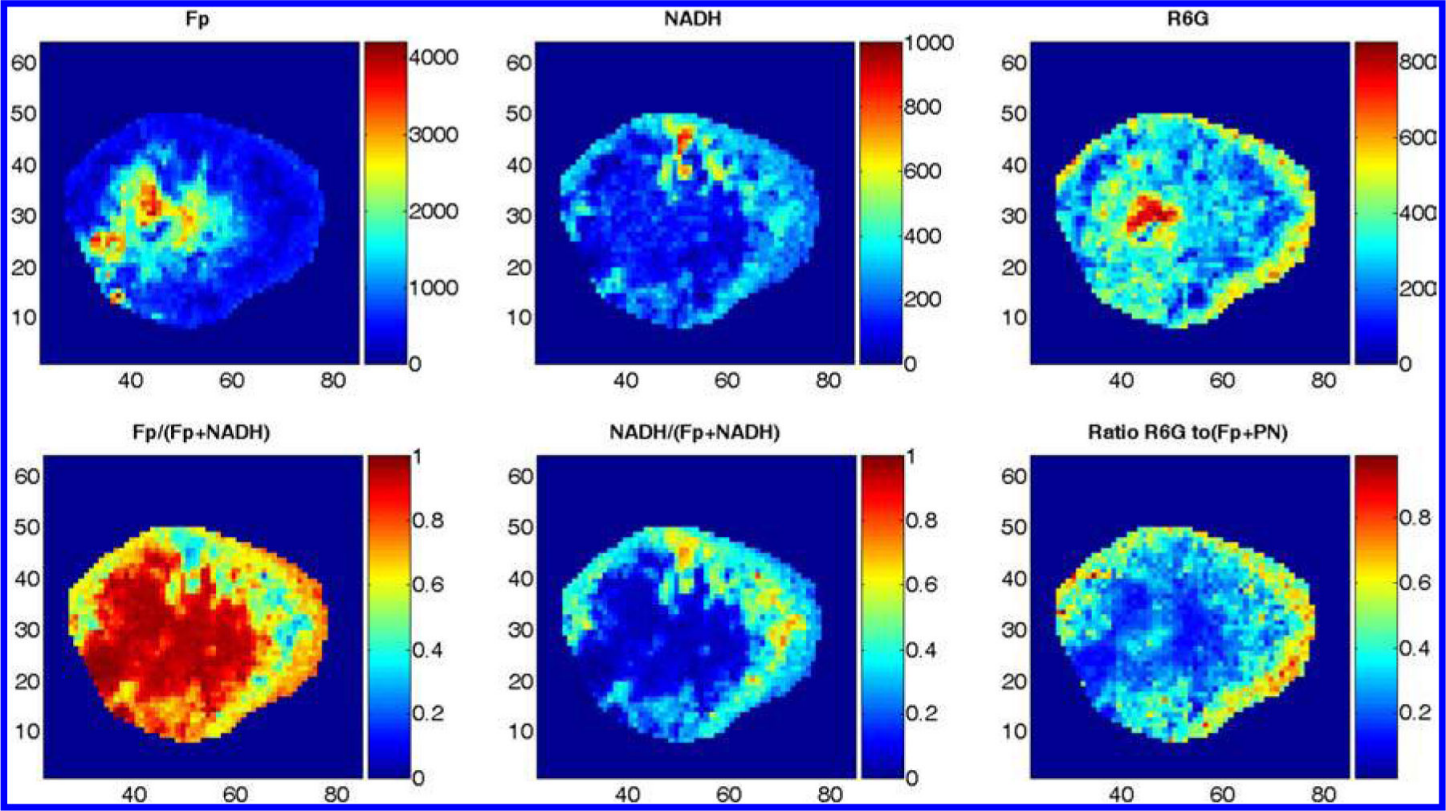
The typical redox and ROS (labeled with R6G) images of a MDA-MB-231 tumor section. R6G stands for rhodamine 6G which was converted from exogenous dihydrorhodamine 6G bound to mitochondria and oxidized by ROS.

Our redox scanning results on melanomas and breast tumors seem to suggest that tumor metastatic potential is associated with more oxidized redox state. Among many of the factors that drive tumor metastasis, high concentration of reactive oxygen species (ROS) have been suspected and is shown to play a critical role. However, demonstrating such mechanism at tissue level with spatial and temporal details is certainly much more difficult than doing it *in vitro.* We injected optical dyes (such as dihydrorhodamine 6G) via tail vein to tumor-bearing mice. Dihydrorhodamine 6G is not charged and thus is more evenly distributed in the cells, whereas rhodamine 6G is cationic and accumulates into the mitochondria. Upon the oxidation by ROS, the nonfluorescenct dihydrorhodamine 6G becomes rhodamine 6G which emits red fluorescence. The fluorescence signals of Fp, NADH, and rhodamine 6G were simultaneously collected from the snapfrozen tumors by employing the Chance redox scanner. We observed the interesting spatial distribution patterns of rhodamine 6G from the MDA-MB-231 tumors (Fig. 4). The interpretation of the data is still rather qualitative and is limited by a few factors. Due to inhomogenous perfusion, the dye concentration in the tumors may be non-uniform and can be very low for the less perfused tumor core. However, in a small area of the central region, rhodamine 6G signal is much stronger than that in the peripheral area. This suggests that there was a higher production of ROS by each cancer cell in this region because the cell density in the central region is usually lower than that of the periphery (unpublished data). ROS is known to drive cancer progression by generating more mutations in DNA and directly activating certain signaling events that facilitate metastasis.^74^ The observation that the tumor cells in the oxidized redox core may have more ROS appears to suggest a plausible mechanism for understanding the correlation between the tumor aggressiveness and the redox imaging indices. The redox state might regulate genetic pathways and drive tumor progression to metastasis via ROS.^75^

On the other hand, active reprogramming by mutated oncogenes and tumor suppressors may yield altered metabolism and this is proposed as a core hallmark of cancer.^61^ It has been known that p53, an important tumor suppressor, regulates mitochondrial respiration and glycolysis as well as many other metabolic pathways in tumors.^76–78^ The inactivation of p53 suppresses mitochondrial respiration and enhances glycolysis as well as increase ROS production and inhibit apoptosis.^79^ Some evidence has already shown that loss of p53 increased colon tumor malignancy.^80^ Loss of p53 in tumor surrounding tissue can generate an inflammatory microenvironment that promotes colorectal tumor cells invasion and lymph node metastasis.^81^ We were interested in whether tumor suppressor p53 regulates the tumor redox state. With a right colon cancer model, we expected to see the p53 status difference reflected in the mitochondrial redox state and the loss of p53 correlates with the more oxidized redox state.

**Fig. 5.**
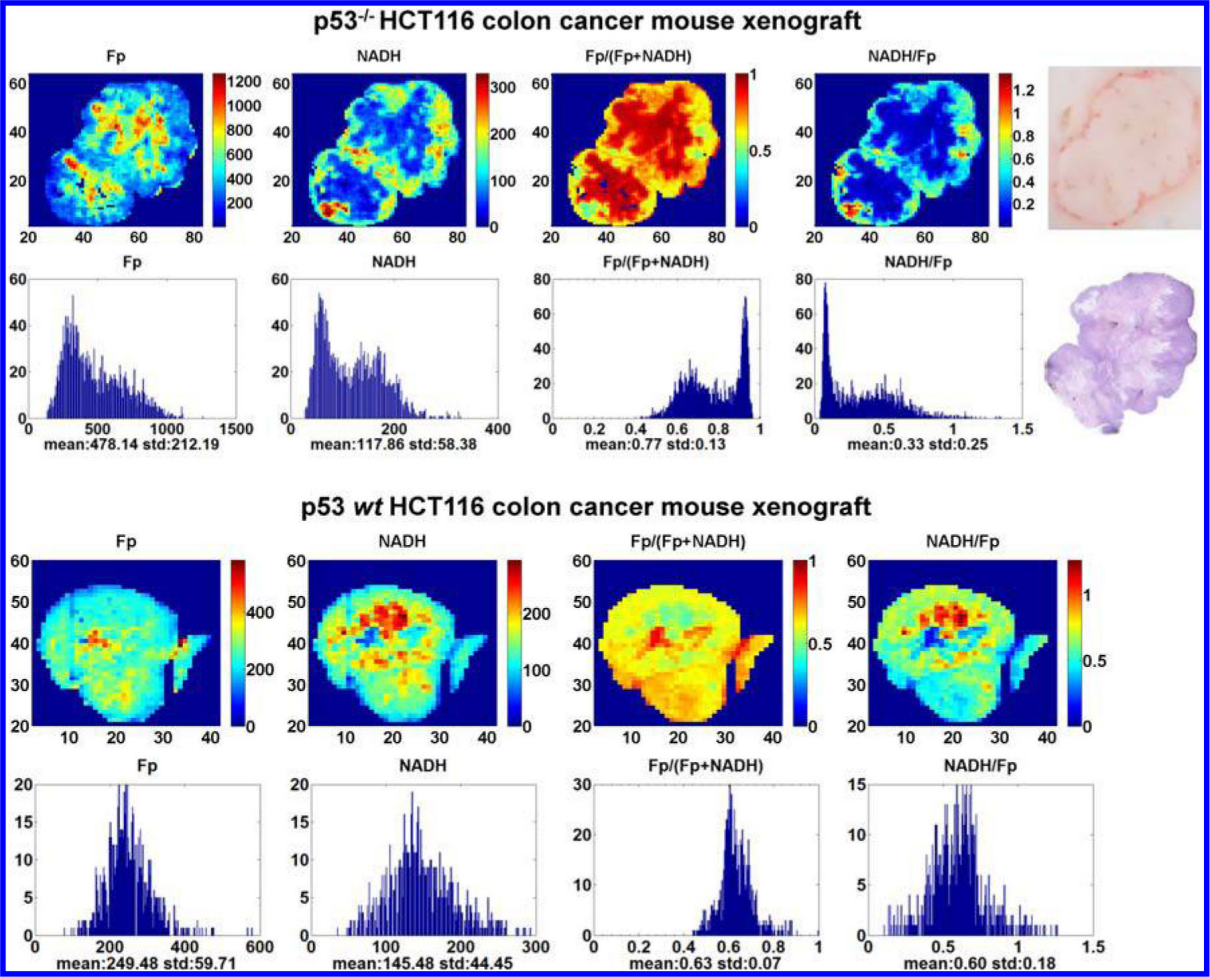
The typical redox images of the p53 wt and p53^–/–^ colon tumors and the corresponding histograms. A photo and the photo-stitched H&E staining image of the p53^–/–^ tumor section were also included. The blue color patterns in the H&E staining appear to correlate with that of the NADH and NADH/Fp images. However, even in the eosin-stained regions, islands of viable cells are found. Also unusual is that the p53 *wt* tumor has some relatively more reduced redox cores (the red patches in the NADH/Fp image), which is not commonly seen for metastatic tumors. The figure is reproduced with permission from Ref. 45.

We obtained two variants of a metastatic colon cancer cell line HCT116^82^ from Dr. Wafik’s lab, one with wild type p53 (p53 *wt*) and the other with p53-null (p53 ^–/–^). The two lines were concurrently and subcutaneously implanted in individual athymic nude mice on both flanks of the body, one side for the p53-null and the other side for wild type.^45^ We found that tumor size played a significant role in the mitochondrial redox state. The larger p53^–/–^ tumors had higher Fp signals and were more heterogeneous in the spatial distribution of the redox state. Additionally the larger ones were also more oxidized than the smaller ones and had more oxidized redox core. After excluding the size effect (p53^–/–^ tumors grow faster), we showed that the p53^–/–^ tumors had higher degree of heterogeneity of the Fp redox ratio and less NADH signal and their redox core was significantly more oxidized than the p53 *wt* tumors^45^ (Fig. 5). These results support the hypotheses that the p53 null mutation may result in a modified redox state and the higher metastatic risk is positively associated with the degree of oxidization as measured by the Fp redox ratio.

**Fig. 6.**
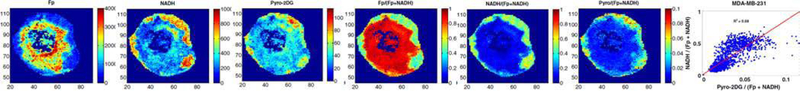
The typical redox images of a MDA-MB-231 aggressive breast tumor section and the glucose (Pyro-2DG) uptake. After normalizing to Fp+NADH to minimize the density variation, the glucose intake pattern is seen to positively correlate with the NADH redox ratio. The figure is reproduced with permission from Ref. 53.

It is worth noting that glucose uptake measured by FDG-PET failed to distinguish these two lines both *in vitro* and *in vivo,* and the CT scan image did not show difference, either.^83^ Because the imaging data were analyzed using global averaging method, the possible difference in the intratumor heterogeneity was ignored. Also, due to the relatively low spatial resolution (~ 2 mm), the FDG-PET technique may not effectively detect the spatial heterogeneity in tumor glucose uptake. Our glucose uptake study showed that the coarseness of glucose uptake heterogeneity in MDA-MB-231 tumors was on the order of 1 mm or less^53,73^ (Fig. 6). The study on glucose uptake by others showed a submillimeter heterogeneity scale in FaDu tumors.^52^ This is beyond the resolving ability of a current PET animal scanner of ~ 2 mm spatial resolution.

#### 3.1.4. The redox state of premalignant tissue

**Fig. 7.**
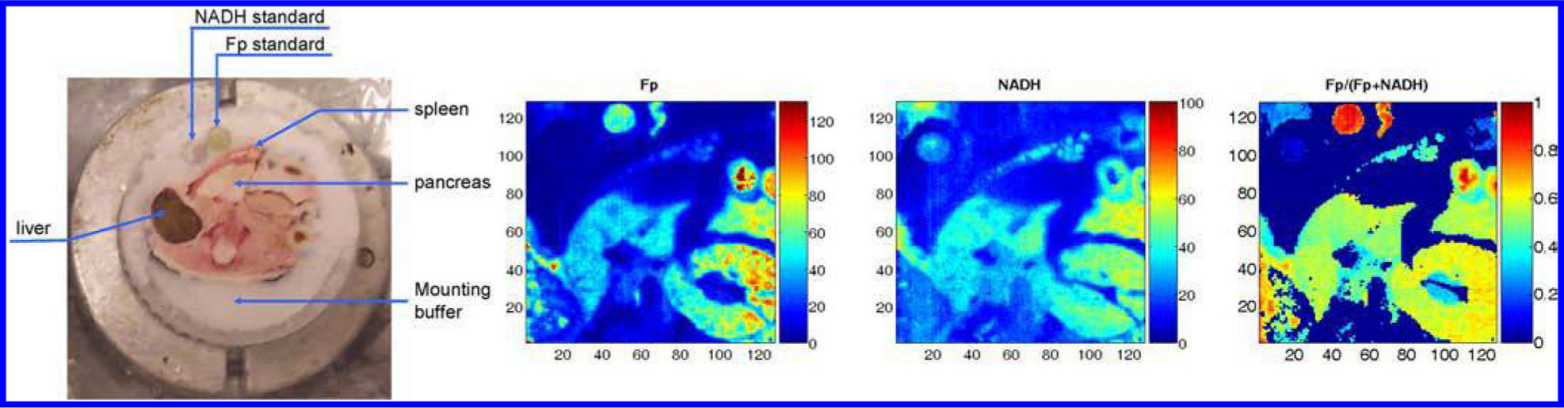
First redox image of different mouse organs.

In the summer of 2008, returning from Singapore, Dr. Chance became very interested in studying the redox state of pancreas. Snap-frozen mice with specific genetic mutation in beta-cells were shipped in from Singapore. However, none of us could recognize where pancreas was located in the frozen mouse body. Dr. Chance called Dr. Matschinsky for help. Accompanied by four of us (Dr. Matschinsky, Dr. Nioka, and the authors), Dr. Chance travelled from the Anatomy Chemistry building to the Stemmler Hall in a wheel chair. Dr. Matschinsky taught us to first look for the spleen because it was easily spotted due to its red color and the pancreas (white color) should be wrapped by the red spleen perpendicularly. That was easy except that it was impossible to only take out the frozen pancreas from the frozen mouse body. We had to use a handsaw to excise a section of the mouse body ~ 2 cm thick that contained the pancreas and the tissues of other organs. Due to the brittle nature of frozen tissues, only one of the wild type pancreases was successfully sectioned out and the mutated ones became unrecoverable. Nevertheless, we obtained our first redox image of mouse organs including spleen, pancreas, kidney, and liver (Fig. 7).

Dr. Chance then initiated a collaborative project with Dr. Ben Stanger who provided us a transgenic mouse model with premalignant pancreas. The mouse model has pancreas-specific PTEN-deletion resulting in infrequent malignant transformation. One of the key alterations in cancer is the activation of the PI3K-PTEN-AKT signal transduction pathway.^59–60–79^ The activation of PI3K/AKT pathway promotes cancer cell survival, proliferation, and transformation. PTEN is a negative regulator of PI3K. The activation of PI3K pathway is also found in pancreatic cancer.^84–85^ By studying the PTEN-null pancreas, we expected to identify certain redox imaging biomarkers for predicting pancreatic premalignancy.

Learning from our previous experience we knew we should not snap-freeze the entire mouse for harvesting visceral organs. The alternative was to anesthetize the animal and perform open chest surgery to resect the needed organs followed by immediate snap-freezing with liquid nitrogen. This method does not introduce detectable metabolic difference on the basis of the experience of another research group.^86^ Dr. Chance again enthusiastically travelled with us to Dr. Stanger’s lab to observe Ben’s skillful surgical demonstration on pancreas removal and was convinced that we could do it the same way for our study.

**Fig. 8.**
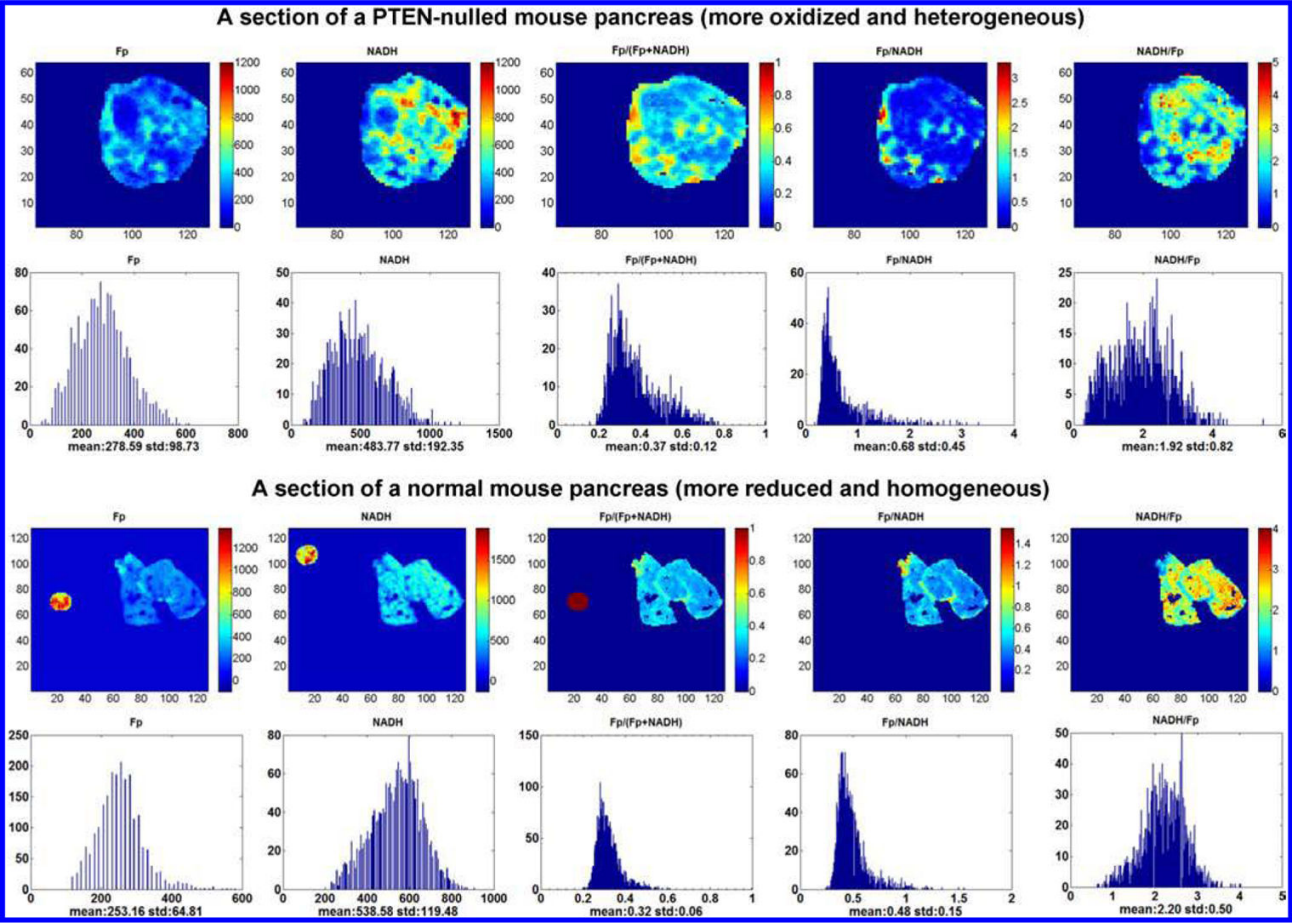
The typical redox images and their corresponding histograms of the PTEN-null pancreas (top two rows) and the normal counterparts (bottom two rows). The figure is reproduced with permission from Ref. 58.

Using the PTEN-null model we were able to show that the PTEN-nulled pancreases are significantly more heterogeneous in the redox state compared to their normal counterparts. In the beginning, we were puzzled at the global averaging results which failed to show statistical difference in the redox indices between the two groups. However, they had to be different because it was so obvious that the mutant tissues had much wider histograms of the Fp redox ratio (Fig. 8). After a few rounds of discussion on the results, we realized that redox state heterogeneity must be taken into account. We used the standard deviation of the Fp redox ratio as the discriminating parameter. The two groups were successfully distinguished by the degree of heterogeneity. Assisted with a big magnifier, Dr. Chance carefully read through and revised the manuscript of our results, which was readily accepted with no need of modification.^46^ As we progressed more with heterogeneity analysis, we adopted curve fitting approach to fit the histogram of the Fp redox ratio to two Gaussian functions and quantified their line widths as a heterogeneity index, which expectedly resulted in consistent outcome with the SD approach. We also used the tissue depth as a covariate for the univariate statistical analysis (SPSS) to take into account the signal variations along the depth. As a result, the two groups differed not only by their degree of heterogeneity but also by the redox state. The PTEN-nulled ones were ~ 20% more oxidized than the control.^58^ To our knowledge, there has not been any report showing if and how the mitochondrial redox state is associated with the abnormality in the PI3K/Akt signaling pathway. Our study is the first investigation at tissue level that linked the mitochondrial redox state to the hyperactive PI3K/Akt pathway and to the cancer transformation.

#### 3.1.5. Assessing the therapeutic effect

Metabolic biomarkers for monitoring therapeutic effect are desired in the clinic. Metabolic changes are expected to occur much earlier before tumor shrinkage. Since the mitochondria play central roles in cellular energy metabolism and trigger apoptotic events by releasing cytochrome c, we hypothesized that cancer drugs, particularly the mitochondriatargeted ones would alter the mitochondrial redox state. We used DLCL2 lymphoma mouse xenografts as the model systems to examine the effect of one cycle (five days) of CHOP treatment on lymphomas by redox imaging.^87–88^ The results showed that the CHOP treatment decreased Fp signals and shifted the lymphomas to a slightly more reduced mitochondrial redox state. The redox state of the CHOP-treated lymphomas also became less heterogeneous. However no significant change was observed in tumor volume on day 5 in the treated group. The observed reduction in the mitochondrial redox state indicates that CHOP administration might have suppressed the mitochondrial metabolism and the tumors became less aggressive. These results suggest that the mitochondrial redox state may respond to the therapeutic strategies earlier than tumor regression response. More thorough studies are needed to investigate whether the redox state may provide an early biomarker for cancer treatment response in general.

### 3.2. Redox imaging of normal tissues/cells

#### 3.2.1. The redox indices detected embryonic stem cell differentiation

**Fig. 9.**
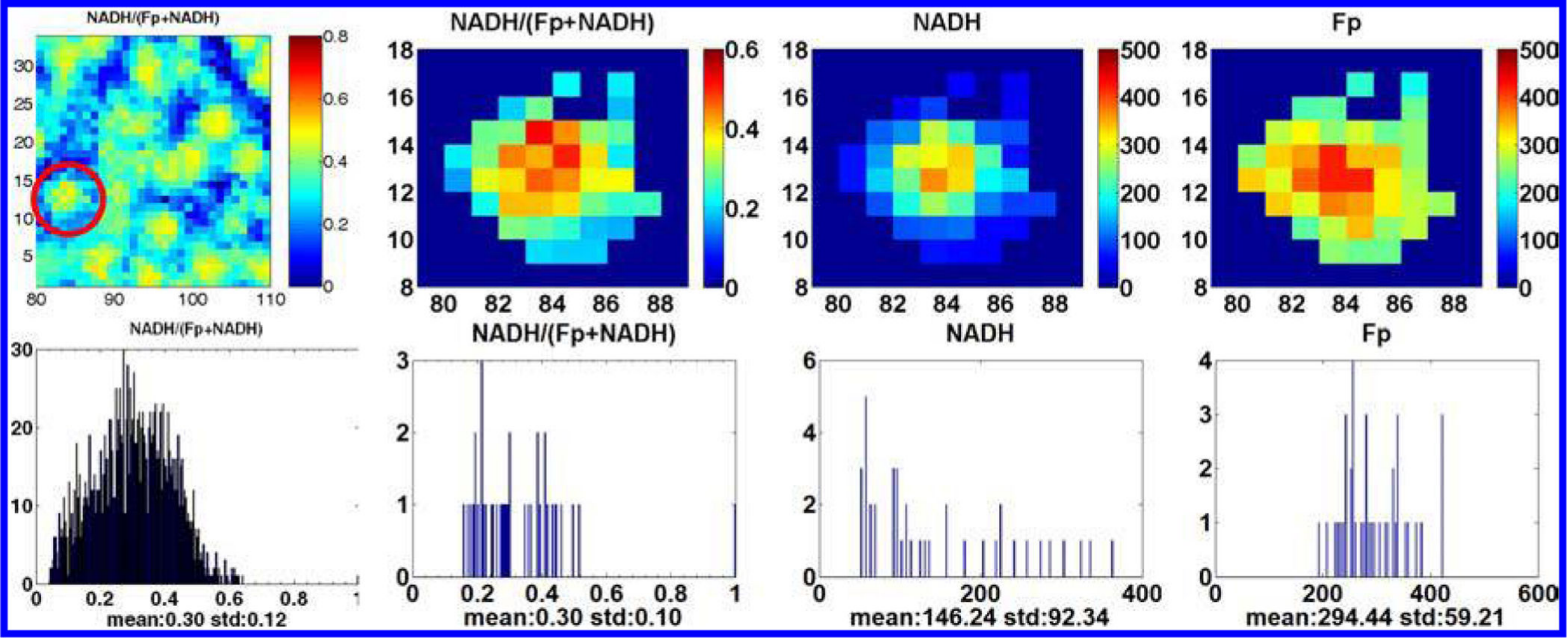
The typical images of mouse embryonic stem cell colonies and their corresponding histograms. The images show that the undifferentiated cells (in the central region) are more reduced. The right three images and the corresponding histograms correspond to the individual ESC colony in the red circle of the first redox image.

In the summer of 2009, coming back from Taiwan, Dr. Chance initiated the embryonic stem cell project and we started the collaboration with Dr. Gearhart’s lab. Dr. Chance suspected that the embryonic stem cells in different differentiation stage might be in different mitochondrial redox state, which could be a biomarker for sorting out the pluripotent stem cells. He questioned how one would know the stem cells injected to the patients were truly pluripotent ones rather than already differentiated ones. Dr. Russell Addis and Davida Goings in Dr. Gearhart’s lab provided us with mouse embryonic stem cells (mESC) colonies. The colonies typically had a size of 200–440 μm and were cultured with feeder cells on round cover slips. We had not scanned the cells grown on a cover slip before. It took some effort to figure out how to mount the cover slip for scanning. We were also concerned about whether the Chance redox scanner could generate interpretable images of these colonies because the lowest resolution achievable by the scanner is 50 *μm* (the bore size of the emission fiber). Including Dr. Chance, we were all delightedly surprised to see the scanning result. The NADH redox ratio image of each individual colony clearly gives a more reduced core in the colony center and a more oxidized rim pattern as shown in Fig. 9, quite opposite to that of the aggressive tumors which have more oxidized core and more reduced rim. By observing the cell morphology, we also saw that the peripheral cells in the colonies had the morphological features of the differentiated stem cells. To confirm this, Dr. Addis stained the colonies with Oct4 antibody which is a pluripotency marker for stem cells. The Oct4 spatial distribution pattern positively correlates with that of the NADH redox ratio, indicating the more pluripotent stem cells are in the more reduced state.

Our result suggests that the mitochondrial redox state could be a stemness indicator of ESCs and could be used as a potential new biomarker for sorting out the ESCs. Dr. Chance was very excited and requested us to phone him while he was in Taiwan. He suggested altering the redox state using the ETC inhibitors, such as cyanide to inhibit OXPHOS, which could be done in future. We published the preliminary results in the special issue of JIOHS in 2011 in memory of Dr. Chance.^89^

It was shown that mitochondrial proliferation is initiated when ESCs start to differentiate spontaneously.^90^ We observed that the differentiated cells had lower NADH redox ratio. A lower NADH redox ratio indicates a more oxidized state and higher mitochondrial activity (State 3). The redox change in the rim of the ESC colonies we observed is consistent with their mitochondrial proliferation. It was also reported that enhanced pluripotency occurred when Complex III in the mitochondria of the human ESCs was inhibited.^91^ It is known that inhibiting ETC results in an increase in NADH and a more reduced state. The cells in the core region of the mESCs were more reduced as observed in our study indicates that these cells were more pluripotent or had more stemness. This is in agreement with the reported pluripotency enhancement by ETC inhibition.

#### 3.2.2. The redox indices of the heart responded to fasting

Using a rat model, we also investigated how the redox indices of the heart responded to fasting. 14.5 h overnight fasting lowered NADH content by ~ 28% in the heart tissue of the rats and the heart became more homogeneous in NADH and Fp distribution. Additionally, we also obtained the 3D heart redox mapping by scanning through the entire heart section by section. We believe this is the first report that published such detailed 3D redox images of the heart (see Xu *et al.* in the same issue).

## 4. Discussion and Future Directions

We have observed significant heterogeneity in the tissue/cell redox indices (NADH, Fp and the redox ratio) with redox scanning under various normal and cancerous conditions. We demonstrated that the tissue redox indices can predict the aggressiveness and/or metastatic potential in animal models. Compared to normal tissues higher lactate generation (the Warburg effect) is a common feature of cancer cells and has been widely studied. However, it is still unclear if and how the Warburg effect is related to the tumor metastatic risk. Some studies have revealed that lactate levels in primary lesion predicted metastatic risk in human cervical cancers and head and neck cancers.^92–94^ Some other studies showed that the highly metastatic breast cancer line MDA-MB-231 generates less lactate than the relatively indolent line MCF-7.^95–98^ Higher lactate level was also shown to correlate with the growth rate of hepatoma.^99^ It is therefore likely that higher lactate production or the Warburg effect may be associated with the high rate of tumor proliferation and growth rather than the tumor metastatic risk. Nevertheless, the redox indices successfully predicted metastatic risk of the tumor xenografts.

One question is whether the redox heterogeneity patterns reflect the variations in cell and/or mitochondrial densities or the variations in cellular metabolic state. Due to the nature of the ratiometric approach, the redox ratio should be less sensitive to cell/mitochondrial density than NADH and Fp. From the typical H&E-stained tumor tissue sections, we know that cell density in the redox core usually is lower than that in the redox rim. Although it remains to be investigated how the mitochondrial density distributes in the tumor models we used, it is likely that the mitochondrial density distribution grossly matches the cell density distribution across a tissue section. Although lower cell density is usually found in the redox cores of metastatic tumors (consistent with lower NADH signals in these areas), the Fp values are higher in the redox cores, indicating a more oxidized intracellular redox state. The exact origins of this high Fp signal in the redox cores (often considered necrotic regions on the H&E-stained slides) are not clear (see discussion below). We have also observed variations in the NADH, Fp, and the redox state within the redox rim or within the redox core, which is likely not reflected in the histology. In future, more work should be done to investigate the origins of tissue metabolic heterogeneity. For example, we can develop a texture analysis method to address the metabolic heterogeneity and we may quantitatively analyze the texture features of the H&E-stained sections and correlate them to that of the redox imaging.

In addition to NADH and Fp molecules, there are other intrinsic fluorescent molecules that may contribute to the autofluorescence signal background but may not be linked to cell metabolism. The signal contribution from tryptophan and collagen can be excluded or minimized by choosing the proper optical filters. Lipofuscin is one of the suspects that may interfere with our interpretation of Fp signals under certain circumstances because of its spectral characteristics^100^ which was also concentration-dependent.^101^ We are not certain whether the Fp signals we collected from tissue samples contain contributions from lipofuscin. Since lipofuscin accumulates in lysosomes, we can in future stain lipofuscin-containing organelles with optical dyes such as lysotracker to see if and how much lysotracker colocalizes with Fp using two photon imaging. We may also try to disturb tissue metabolic states and separate the metabolic sensitive NADH or Fp signals from the insensitive background signals. Nevertheless, our current data indicate that these autofluorescence-based imaging indices and their associated standard deviations can be developed as the quantitative biomarkers for discriminating between precancer/ cancer and normal tissues or predicting tumor risk.

The redox scanning technique may also be applied to studying the developmental processes and other diseases such as mitochondrial diseases, diabetes, and neurodegenerative diseases. Since redox scanning is invasive and not suitable for *in vivo* applications, we have also been developing non-invasive NMR methods for imaging the tissue redox state *in* vivo.^102–103^ Readers are referred to the related references since the content is beyond the scope of this review.

## 5. Summary

**Fig. 10.**
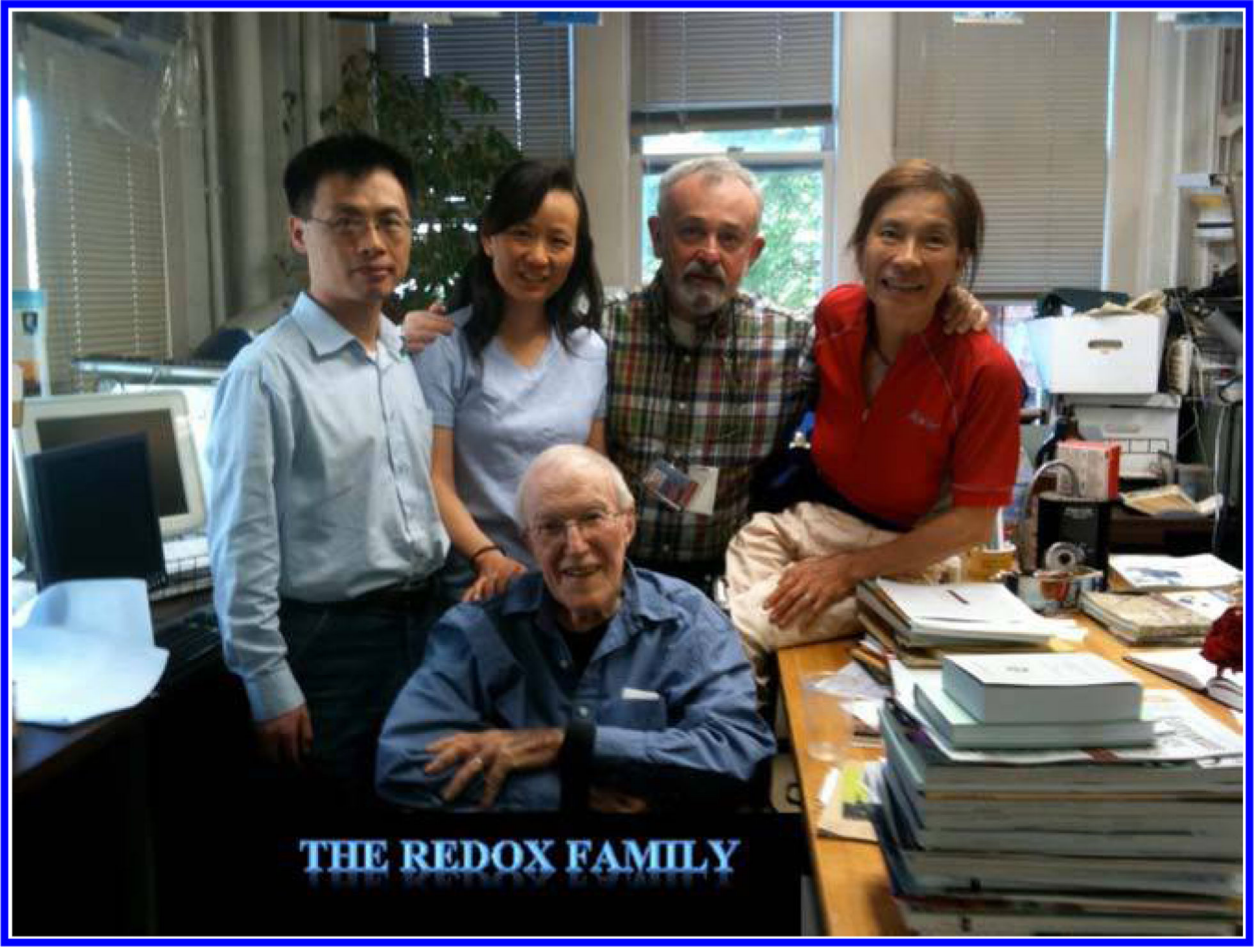
Photo of Dr. Chance with other key members of the redox team (Taken after a pleasant research discussion on 7th July 2010 when Dr. Chance was approaching his 97th birthday. From back left: Lin Z. Li, He N. Xu, Jerry D. Glickson, Shoko Nioka).

In this paper, we briefly reflect on our collaboration with the late Dr. Chance and summarized the research achievements employing the Chance redox scanner since 2005. With his active support to and participation in our redox team (Fig. 10), we discovered that the redox indices and their standard deviations were associated with tissue abnormality, tumor metastatic potential, tumor p53 status, PI3K pathway activation, the therapeutic effect of cancer drug, embryonic stem cell differentiation, and fasting. Together, our work has demonstrated a good potential of developing certain quantitative redox imaging biomarkers for studying tissue metabolic state and the heterogeneity in both normal and pathological tissues.
